# The Medicinal Chemistry of Artificial Nucleic Acids and Therapeutic Oligonucleotides

**DOI:** 10.3390/ph15080909

**Published:** 2022-07-22

**Authors:** Miklós Bege, Anikó Borbás

**Affiliations:** 1 Department of Pharmaceutical Chemistry, University of Debrecen, Egyetem tér 1, 4032 Debrecen, Hungary; bege.miklos@euipar.unideb.hu; 2 Institute of Healthcare Industry, University of Debrecen, Nagyerdei körút 98, 4032 Debrecen, Hungary; 3 MTA-DE Molecular Recognition and Interaction Research Group, University of Debrecen, Egyetem tér 1, 4032 Debrecen, Hungary; 4 National Laboratory of Virology, University of Pécs, Ifjúság útja 20, 7624 Pécs, Hungary

**Keywords:** oligonucleotides, phosphorothioate, phosphorodiamidate morpholino oligomers (PMOs), gene silencing, antisense, RNA interference, aptamer, splicing modulation, mRNA vaccine

## Abstract

Nucleic acids play a central role in human biology, making them suitable and attractive tools for therapeutic applications. While conventional drugs generally target proteins and induce transient therapeutic effects, nucleic acid medicines can achieve long-lasting or curative effects by targeting the genetic bases of diseases. However, native oligonucleotides are characterized by low in vivo stability due to nuclease sensitivity and unfavourable physicochemical properties due to their polyanionic nature, which are obstacles to their therapeutic use. A myriad of synthetic oligonucleotides have been prepared in the last few decades and it has been shown that proper chemical modifications to either the nucleobase, the ribofuranose unit or the phosphate backbone can protect the nucleic acids from degradation, enable efficient cellular uptake and target localization ensuring the efficiency of the oligonucleotide-based therapy. In this review, we present a summary of structure and properties of artificial nucleic acids containing nucleobase, sugar or backbone modifications, and provide an overview of the structure and mechanism of action of approved oligonucleotide drugs including gene silencing agents, aptamers and mRNA vaccines.

## 1. Introduction

Nucleosides are essential for every known form of life and their derivatives play important roles in numerous biological processes. Cyclic nucleoside-monophosphates (e.g., cGMP, cAMP) are involved in signal transduction, adenosine-triphosphate is the main energy storage molecule of cells, adenosine-diphosphate is a platelet activator, nicotinamide-adenine-dinucleotide (NAD), nicotinamide-adenine-dinucleotide-phosphate (NADP) and their reduced forms are crucial cofactors in vital cellular redox reactions. There are also nucleoside-type secondary metabolites that possess antibiotic activity, e.g., uridyl peptides including pacydamycines [1].

However, the most important nucleoside derivatives are undoubtedly the nucleoside-5′-phosphate esters, called nucleotides, and their polymers, nucleic acids (NAs). In nucleic acids, nucleotides are linked through a 3′,5′-phosphodiester bond. The two main types of NAs are deoxyribonucleic acid (DNA) and ribonucleic acid (RNA).

The different types of RNA have different functions. Messenger RNA (mRNA) is responsible for transferring genetic information from the nucleus to the cytoplasm. mRNAs are synthesized through a process called transcription, and then undergo various modifications such as capping (incorporation of a 7-methylguanosine unit at the 5′-end), polyadenylation (synthesis of a polyA tail at the 3′-end), and splicing (cutting out introns from the premature RNA). The role of transfer RNA (tRNA) is to deliver amino acids (AAs) to the ribosome. tRNA contains a wide variety of modified nucleobases e.g., hypoxanthine, various 5-modified uracils, etc. [2,3,4]. Ribosomal RNA (rRNA) is a component of the ribosome, the cellular organell of translation [5]. Micro-RNA (miRNA) is an interesting type of RNA that has a regulatory function. The miRNA has a sequence that is complementery to a short segment of the target mRNA, thereby allowing it to direct the RNA-induced silencing complex (RISC) to the target mRNA, which causes inhibition of translation. The miRNA is synthesized by RNA polymerase II, first producing a primary miRNA (pri-miRNA) that has a partially double-stranded structure. Primary miRNA is cleaved by the DROSHA enzyme complex to produce a precursor-micro RNA (pre-miRNA) with a hairpin structure. Pre-miRNA is transported to the cytoplasm by exportinV, where Dicer, an endoribonuclease, cleaves the hairpin part of the molecule. The resulting mature double-stranded miRNA is incorporated into the RISC, then one of the two strands is degraded, and the other performs the control function. The role of miRNA is to regulate the gene expression of the cell [6]. Small interfering RNA (siRNA) is similar to the miRNA, but its function is to protect the cell from exogenous NAs, for example viral nucleic acids [7]. Small activating RNA (saRNA) can hybridize to the promoter region of the target gene in order to enhance gene expression [8]. There are also RNAs with catalytic activity, called ribozymes [9].

DNA has one simple, but crucial function: to store the genetic information of an organism. There are two important differences between DNA and RNA. First, DNA contains 2-deoxy-D-ribose in its building blocks instead of D-ribose. This increases the half-life of the molecule, which is necessary for the DNA to reliably store the genetic information. Second, DNA contains thymine (5-methyluracil) instead of uracil. The reason for this is that cytosine can be converted into uracil by spontaneous deamination and this phenomenon would pose an insuperable difficulty for repair mechanisms if uracil were a regular nucleobase in DNA. The reduction of the 2′-OH group and the incorporation of the CH_3_ group into the 5-position of uracil requires resources and energy. In the case of RNA, the body can omit these energy-intensive modifications because the half-life of RNA is shorter, therefore, it is less affected by the cytosine-uracil conversion. In addition, due to the continuous flux in the cell’s RNA pool, some errors in the RNA have no serious consequences [10].

**Figure 1:**
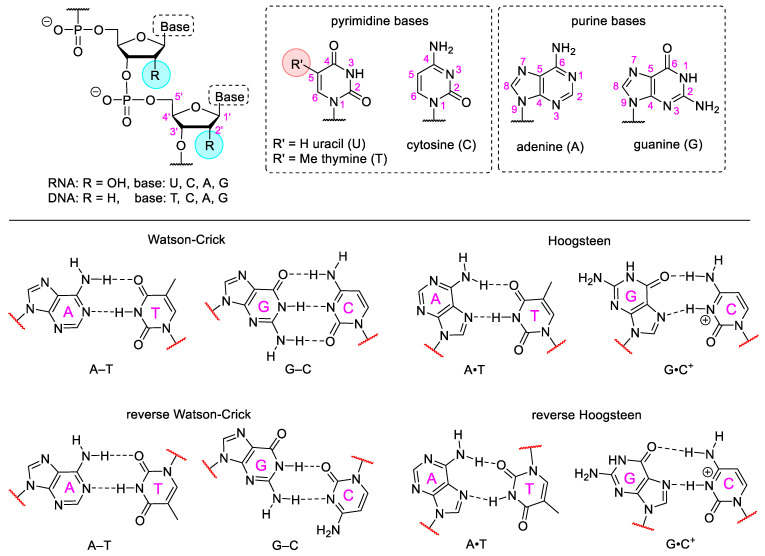
The structure of RNA and DNA and Watson–Crick and Hoogsteen base pair systems.

The nucleobases contain H donor (NH_2_ or NH) and H acceptor (non-binding electron pairs of N and O) groups at certain positions, which makes them suitable for forming H-bonds with each other. Hydrogen bonds are the strongest intermolecular binding forces and one nucleobase can form multiple hydrogen bonds, allowing NAs to form stable duplexes with complementary NAs. Nucleobases can form base-pairs in a variety of patterns. The most important is the canonical Watson–Crick base pairing, in which the base pairs are adenine-thymine (A-T) and guanine-cytosine (G-C). There are 2 H bonds between A and T and 3 H bonds between G and C. In the case of the A-T pair, the H donor positions are T-3-NH and A-6-NH_2_, while the H acceptor groups are T-2-C=O and A-1-N. For the G-C pair, the H donor positions are G-2-NH_2_, G-1-NH and the C-4-NH_2_ groups, while the acceptors are G-6-C=O, C-2-C=O and the C-3-N (Figure 1). Another significant, but less common base pairing system is the Hoogsteen system. Similarly to the Watson–Crick system, A·T and G·C pairs are formed, but different groups are involved in the formation of the H bonds. Another important difference is that cytosine must be protonated at the 3N position to be able to take part in the Hoogsteen pairing [11,12].

**Figure 2:**
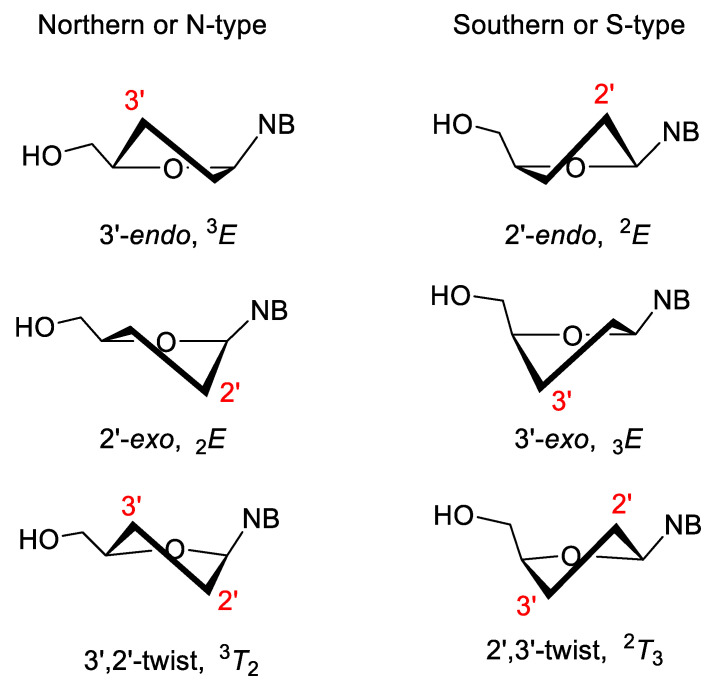
Sugar puckering in nucleic acids.

The furanose ring of the nucleoside can exist in several conformations, this is called “puckering”. Some of the possible conformers are shown in Figure 2. If four of the five atoms in the ring are in one plane and the fifth is above that plan, it is called the *endo* conformation, while the conformer in which the fifth atom is below the plane is called the *exo* conformation. The 3′-endo conformations are called the northern or N-type, while the 2′-endo conformations are known as the southern or S-type conformations. S conformations are more common in B-DNA, but N conformations are predominant in A-DNA and in RNA [13,14,15].

Due to the central role of nucleic acids in human biology, they are suitable and attractive tools for therapeutic applications. The next section of this review briefly describes the mechanisms and techniques that underlie the use of nucleic acids as therapeutics, diagnostics and vaccines.

However, natural nucleic acids are not ideal for biological applications, primarily due to their sensitivity to nucleases, which cause short half-life and therefore limit efficiency. For this reason, synthetic nucleic acid analogues, also known as xeno nucleic acids (XNAs), are generally used which have increased stability and more favourable pharmacokinetic properties than their natural counterparts. The main chapter of this review is devoted to the world of xeno nucleic acids, which serve as a rich source for medicinal and technological applications. The vast and extremely diverse array of chemically modified oligonucleotides is impossible to comprehensively present in a single review article. A number of reviews have been published on subareas of the topic, such as delivery [16], metabolism [17], or antisense use [18,19]. Here, we provide a thorough overview of the major types and latest versions of xeno nucleic acids that have been developed to date, briefly addressing the rationale behind the modifications and highlighting the impact of these modifications on physicochemical properties and biological function. Synthetic methods and challenges are not discussed here as they have been reviewed in recent works [20,21].

Finally, we present nucleoside analogue drugs and vaccines approved by the United States Food and Drug Administration (FDA) and the European Medicines Agency (EMA) describing their chemical composition, mode of action, route of administration, as well as their therapeutic efficacy and adverse effects.

## 2. The Use of Oligonucleotides in Therapy and Diagnostics and the Mechanisms behind Them

Today, we are witnessing the widespread use of oligonucleotides, not only in medicines such as drugs, vaccines, and diagnostic tools, but also in various fields of technology. The principles of practical applications in therapy and diagnostics are described very briefly here.

*Polymerase chain reaction* (PCR) is an effective method for detecting very small amounts of NA by multiplying it. It is useful for detecting specific mutations of the NA of a pathogen. For instance, it is the most widely used method for the detection of the SARS-CoV2 infection [22]. The PCR test requires appropriately designed primer oligonucleotides, which can hybridize to the target single-stranded (ss) NA, in order to enable the polymerase enzyme to start the synthesis based on the sequence of the selected NA. PCR techniques can also be used in research or criminology [23,24].

The so-called *decoy oligodeoxynucleotides* (ODNs) have been designed to specifically bind to transcription factors in order to inhibit the expression of the target gene [25].

*Gene editing* or genome editing is a rapidly evolving method for creating new, genetically edited organisms including plants [26], fungi, animals [27] or even humans [28]. The latest and most effective method for gene editing is the Clustered Regularly Interspaced Short Palindromic Repeats (CRISPR)-Cas system, which utilizes the Cas-9 endonuclease, which can create double-strand breaks in the DNA in the sequence where it is complementary to the “guide RNA” of the enzyme. By properly designing the sequence of the guide RNA, it is possible to select the specific segment of the DNA to be cut by the enzyme. Instead of native RNA, artificial oligonucleotides can be used to guide the Cas-9 [29,30].

*Aptamers* are short oligonucleotides, which can specifically recognise and bind to a molecular target [31]. Because binding is not based on base-pair hybridization, the target can be not only a nucleic acid, but also a peptide, protein or small molecule. Aptamers have great potential in diagnostics and in clinical use [32,33].

The *mRNA vaccines* represent a new concept in vaccination. Unlike other types of vaccines (e.g., subunit, or attenuated type), in mRNA vaccines the active ingredient is an mRNA, which encodes the target antigene protein and also contains 5′ and 3′ untranslated regions (UTRs) that are necessary for translation and may affect the rate of protein synthesis. The cells first synthesize the antigenic protein encoded by the mRNA. Then, after post-translational modifications, the antigen appears on the cell surface either in native form, or bound to the major histocompatibility complex I (MHC I), allowing the immune cells to recognize and elicit an immune response. These mRNA vaccines do have significant advantages over conventional vaccines: they are very efficient and safe due to the short half-life of mRNA and its degradation into nontoxic metabolits by natural cellular processes. Another advantage is that they are easy to produce. If the targeted antigene is altered because of the mutation of the pathogen, a new vaccine can easily be developed if the gene sequence of the antigene is known. Disadvantages of mRNA vaccines include instability, immunogenicity of mRNA, and difficulty in entering the cell. However, technological advances have offered solutions to these problems: stability can be increased, while immunogenicity of the RNA can be reduced by synthetic modifications and cellular uptake can be achieved with modern delivery systems [34].

*Gene silencing* was discovered by Zamecnik and Stephenson in 1978 [35]. They observed that replication of Rous sarcoma virus can be inhibited by oligodeoxynucleotides. Gene silencing means the selective and temporary blocking of the expression of a target gene at any point of the process such as transcription, translation, splicing. Gene silencing causes a decrease in the amount of the gene product. The advantage of gene silencing is that it is based on sequence-based interactions between nucleic acids, so it is very specific, and not only inhibits the gene product, but physically reduces the amount produced. However, it works slowly because gene silencing cannot affect previously synthesized proteins and their degradation takes time. In addition, the use of oligonucleotides for gene silencing poses pharmacokinetic, pharmacological, technological and chemical challenges that can be overcome by using modified oligonucleotides instead of native ones. The main methods for gene silencing include RNA interference, anti-gene strategy, antisense strategy and the use of ribozymes.

*RNA interference* was discovered in 1998 [36], its mechanism by endogenous siRNA has been briefly discussed above [7]. It can also be performed by exogenous, double stranded nucleic acids. Translation of the targeted mRNA is blocked sterically, and the mRNA is also degraded by the argonaute protein, which is part of the RISC with RNase activity. The oligonucleotides used are analogues of mature miRNA or pre-miRNA. RNA interference is very effective because the siRNA in the RISC is not degraded, so a single molecule of the active ingredient can assist the cleavage of multiple mRNA molecules [37,38].

During *anti-gene strategy*, the oligonucleotide forms a triplex with the DNA, blocking transcription at either the elongation or initiation stage. Triplex formation is possible on a polypurine tract, since the purine bases can simultaneusly be part of a Watson–Crick and a Hoogsteen base-pair. Unlike thymine, cytosine must be protonated to participate in triplex formation, which requires an acidic pH. It limits the use of the method, as the stability of the triplex is also pH dependent. This problem can be eliminated by replacing the cytosine to pseudoisocytosine. The advantage of this method over RNA interference is that while there are large amounts of mRNA molecules in the cytoplasm, there are only two copies of DNA in the cell, so fewer molecules of the active ingredient may be sufficient. The disadvantage is that it is difficult but necessary to deliver the oligonucleotide to the nucleus. The triplexes formed do not degrade in the nucleus (however, the number of plasmids can be decreased this way) which means that the effect only lasts for the duration of binding [39,40,41].

*The antisense strategy* is the simplest type of gene silencing. The principle is that short (16–20 nucleotide) single-stranded oligonucleotides can bind to the complementer sequences of the mRNA and sterically block transcription or RNA processing, and the double-stranded RNA formed can activate RNAases, resulting in degradation of the target mRNA. Theoretically, any region of mRNA can be targeted, however the efficiency of antisense gene silencing may differ in different sequences due to different accessibility of target regions. Therefore, some oligonucleotides targeting different sequences on the mRNA are tested during antisense drug development. By increasing the length of the oligonucleotide, its binding strength can be increased, but the selectivity is reduced because the sequences that are too long include shorter segments, for example, 11 decameric sequences are found in an icosamer (twentymer) [42,43,44].

*Ribozymes* can be used to cleave target mRNA, thereby inhibiting gene expression [45].

## 3. Artificial Nucleic Acids

The therapeutic use of natural nucleic acids is hampered by their sensitivity to nucleases and their poor pharmacological properties due to their polyanionic nature. These dificulties can be overcome by proper chemical modifications to either the nucleobase, the ribofuranose unit or the internucleotide linkage [46]. Nuclease stability can be enhanced by modifying the phosphodiester backbone and sugar units. Backbone modifications also modulate pharmacokinetic properties including cellular uptake. Sugar modifications may enhance hybridization efficiency, which can be quantitatified by Tm value, i.e., the temperature at which the two strands of duplex are separated. Hybridization selectivity (Tm decrease/mismatch) can be improved by altering the nucleobases or the backbone.

**Scheme 1:**
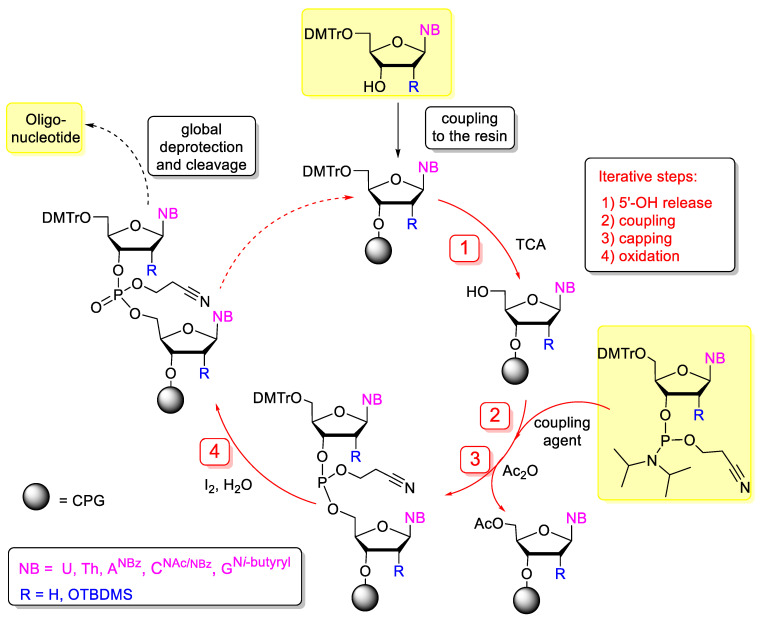
Solid-phase synthesis of oligonucleotides by phosphoramidite chemistry. The iterative steps of synthetic cycle are highlighted in red. DMTr: 4,4′-dimethoxytrityl, TBDMS: *t*-butyldimethylsilyl, TCA: trichloroacetic acid, Ac_2_O: acetic anhydride, CPG: controlled pore glass.

Before discussing the chemical structure and biological properties of XNAs, we briefly describe the process of oligonucleotide synthesis. During biosynthesis, enzymes build up nucleic acids from the 5′-end monomer to the 3′-end. The chemical synthesis of oligonucleotides and artificial nucleic acids—which is generally performed by automated solid phase oligonucleotide synthesis (SPOS)—proceeds in the opposite direction. In this case, the first nucleoside is attached to the solid phase, which is usually a controlled pore glass (CPG), through its 3′-OH group, while the 5′-hydroxyl is protected in form of a 4,4′-dimethoxytrityl (DMTr) ether. Then, the synthesis cycle consisting of four reactions is started. First, DMTr group is cleaved with trichloroacetic acid (TCA) to release the 5′-OH group. In the next step, 3′-*O*-(*N*,*N*-diisopropyl)phosphoramidite derivative of 5′-*O*-DMTr-nucleoside is added together with the activating agent which is an acidic azole catalyst, e.g., 1H-tetrazole or 4,5-dicyanoimidazole. In this coupling step a phosphite triester internucleotide linkage is formed. Subsequently, due to the incompleteness of the coupling, a capping is necessary, which means the protection of the remaining 5′-hydroxyl groups by acetylation with Ac_2_O in order to exclude the unreacted monomers from further synthetic cycles. In the last step of the cycle, the phosphite derivative obtained is oxidized into a phosphate with iodine. The above steps must be repeated n-2 times to obtain an oligomer of n nucleoside in length. Finally, the protecting groups are removed and the complete oligomer is cleaved from the solid phase. Benzoyl group is used to protect the amino groups of the nucleobases in cytidine and adenosine, and *i*-butyryl is used to protect the amino group of guanosine. In RNA synthesis, the 2′-OH group is protected with *tert*-butyldimethylsilyl ether (TBDMS) (Scheme 1) [20,47,48,49]. In addition to the above typical protocol, there are other methods for SPOS. The synthesis of modified nucleic acids may involve other steps or modifications.

### 3.1. Base Modified Nucleic Acids

**Figure 3:**
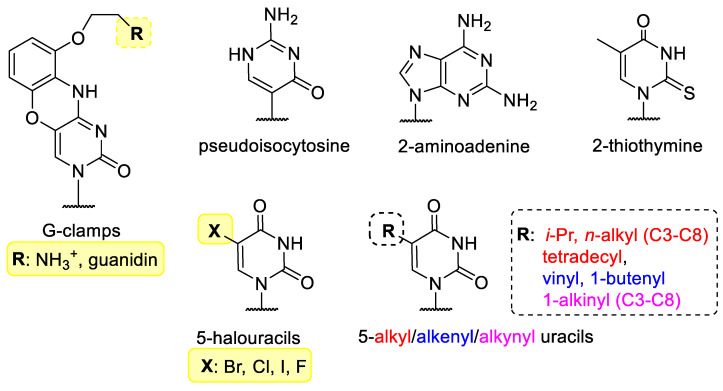
Modified bases in XNAs.

Instead of canonical bases such as thymine/uracil, adenine, cytosine and guanine, other bases can be incorporated into nucleic acids that can can be involved in hibridization (Figure 3). G-clamps (9-(2-aminoethoxy)-phenoxazine derivatives) are tricyclic nucleobase analogues named after the G-shaped device of the same name. They are functionally cytosine analogues, because they can bind to guanine, however G-clamp can form four hydrogen bonds with guanine, instead of the 3 H-bond in the G-C pair. This greatly increases the binding strength of the G-clamp-containing oligonucleotides and may increase the efficacy of hybridization [50,51,52]. Pseudoisocytosine may be part of a Hoogsteen base pair without protonation, therefore pseudoisocytosine-containing oligonucleotides have good triplex-forming potential, for example with DNA during anti-gene gene silencing, therefore, pseudoisocytosine is favourably used in peptide-nucleic acids (vide infra) [53,54].

2-Aminoadenine and 2-thiothymine bind weakly to each other, but can bind strongly to the natural nucleobases [55,56]. The 5 position of pyrimidine nucleobases is not involved in hybridization and is therefore often chosen to incorporate halogens [57] or small alkyl, alkenyl or alkinyl groups [58].

### 3.2. Sugar-Modified Nucleic Acids

**Figure 4:**
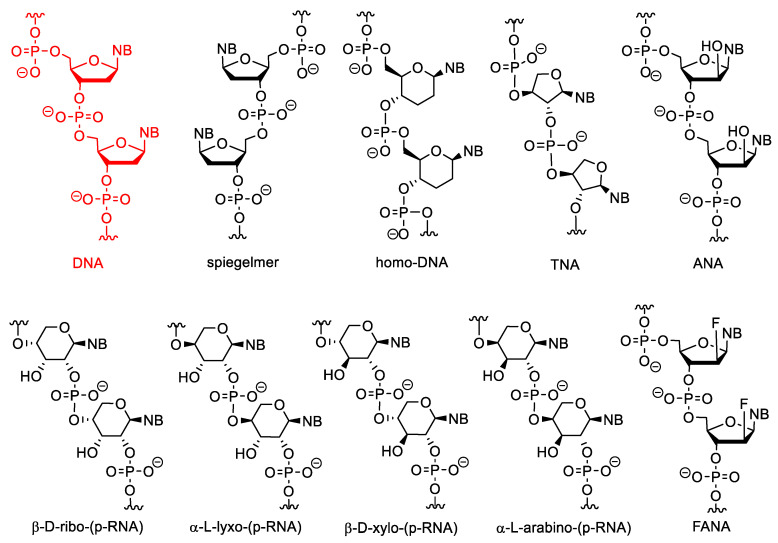
The structure of DNA and XNAs with other sugars than β-D-ribofuranose.

Selected examples for sugar-modified nucleosides are shown in Figure 4. The β-D-ribofuranose unit of natural NAs can be replaced by α or L stereoisomers or other sugars. L-nucleotides are enantiomers of D-forms, therefore their oligomers are called “spiegelmers” (spiegel is mirror in German). Spiegelmers are resistant to nucleases and are not immunogenic [59]. They are not used in gene silencing, but have great potential as aptamers [60].

Nucleic acids may be composed of α-nucleotides, and these derivatives may have strong antisense activity [61,62]. The α and L nucleotides can be combined with other modifications, such as “locking” (see below) [63].

In pyranosyl RNAs (p-RNA), the ribofuranose ring is replaced by pento- or hexopyranoses. These derivatives have been synthesized primarily not for therapeutic purposes but as tools to study the relationships between the structure and biological functions of nucleic acids and thus to better understand the structural criteria of natural nucleic acids [64,65,66,67]. HomoDNA is an NA analogue that contains 2′,3′-dideoxyglucopyranose instead of ribose (altro-, and allopyranose also have been investigated). Base pairs between homoDNAs do not closely follow the Watson–Crick rules, A-A and G-G base pairs can also be formed according to antiparallel reverse Hoogsteen pairing [68].

Pentopyranosyl oligonucleotides (p-RNAs) contain a pentopyranose ring (β-D-ribose, β-D-xylose, α-L-lyxose or α-L-arabinose) as the sugar component, as the name implies. Base-pairing of these derivatives is not only stronger but even more selective than in DNA or RNA. p-RNAs are able to hybridize with each other [69,70,71,72]. TNAs (threofuranosyl nucleic acids) contain α-L-threofuranose instead of β-D-ribofuranose. Unlike β-pyranose nucleic acids, TNAs can hybridize to DNA and RNA. The hybridization strength of TNA duplexes is similar to that of the corresponding DNA or RNA suplexes [73]. HomoDNAs are connected through a 6′→4′ linkage, whereas 4′→2′ and 4′→3′ derivatives were formed in p-RNA. In TNA the phosphodiester bonds are in the 3′→2′ position. In arabinonucleic acids (ANA), the configuration of the C-2′ position is changed. These derivatives can form duplexes with RNA and DNA and activate RNAse H. Interestingly, the conformation of the sugar ring (“pucker”) in these molecules is closer to DNA than to RNA [74]. Their 2′-deoxy-2′-fluoro analogues are FANAs, which form more stable duplexes with RNA and especially with DNA [75].

**Figure 5:**
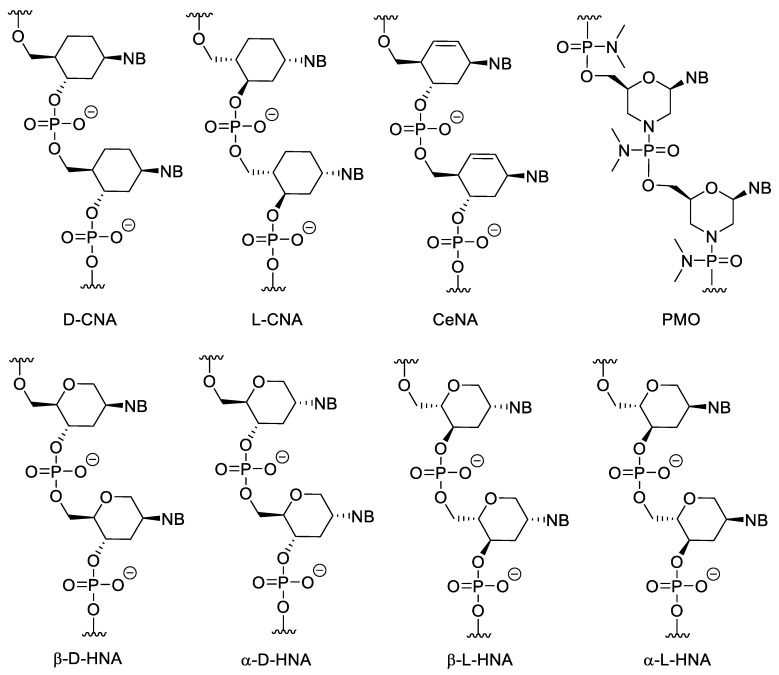
Structure of CNAs, CeNA, HNAs and PMO. PMOs contain both sugar and backbone modifications.

Cyclohexanyl nucleic acids (CNA) contain cyclohexane ring (Figure 5). Both D and L isomers were synthesized and investigated. The Tm of D-CNA/D-CNA and L-CNA/L-CNA duplexes is higher than that of the L-CNA/D-CNA duplexes. Only D-CNA can hybridize to RNA and DNA [76]. Their 5′-6′ unsaturated analogues are the cyclohexenyl nucleic acids (CeNA). Incorporating cyclohexenyl monomers into DNA oligonucleotides, slightly decreases the Tm value of the duplex formed with complementary DNA but increases the Tm of the duplex, formed with complementary RNA [77]. The CeNA-RNA duplexes can activate RNAse H [78]. Hexitol nucleic acids (HNAs) contain 1,5-anhydrohexitol. All four isomers (β-D, α-D, β-L, α-L) were synthesized, however β-D-HNA was the first and most studied [79]. They can hybridize to natural nucleic acids, especially RNA [80] and can be used for gene silencing in antisense strategy [81] or RNA interference [82]. α-D and α-L-HNA can also form duplexes with RNA, while β-L-HNA is unable to do this [79].

Phosphorodiamidate morpholino oligomers (PMOs) contain modifications to both the sugar unit and the backbone. The furanose units are replaced with morpholine rings attached to each other through phosphorodiamidate linkages. The morpholine motif is obtained by oxidizing the corresponding ribonucleoside derivative into a 2′,3′-secodialdehyde with IO_4_^−^ and then forming the nitrogen-containing ring by a reductive amination-cyclization reaction. PMOs have several advantages, such as good water solubility, inexpensive synthesis, high stability in biological systems due to their resistance to many hydrolytic enzymes, good hybridization properties and high specificity [83]. Due to its neutral backbone PMO is less prone to bind to proteins than native NAs, which decreases non-specific, undesirable interactions. Importantly, PMO/RNA duplexes cannot activate RNase H. In gene silencing, this may be a disadvantage because the level of the targeted mRNA is not decreased by enzymatic degradation. However, this property can be exploited. Since the formed duplexes are not cleaved, non-targeted but non-specifically binding nucleic acids do not degrade, which means a higher selectivity. In addition, due to the lack of the degradation, PMOs can be used to modulate the splicing of pre-mRNA, to gain alternatively spliced mRNAs, which can be therapeutically used. Their low cellular uptake can be improved by conjugating them to cell penetrating peptides (CPPs) or guanidin containing moieties [84,85]. PMOs can be used as gene silencing tools against viruses [84]. PMO mediated gene silencing has been experimented in, among other things, zebrafish [86], xenopus [87] and sea urchin [88]. There are a few approved PMO-based drugs on the market today (see more below).

**Figure 6:**
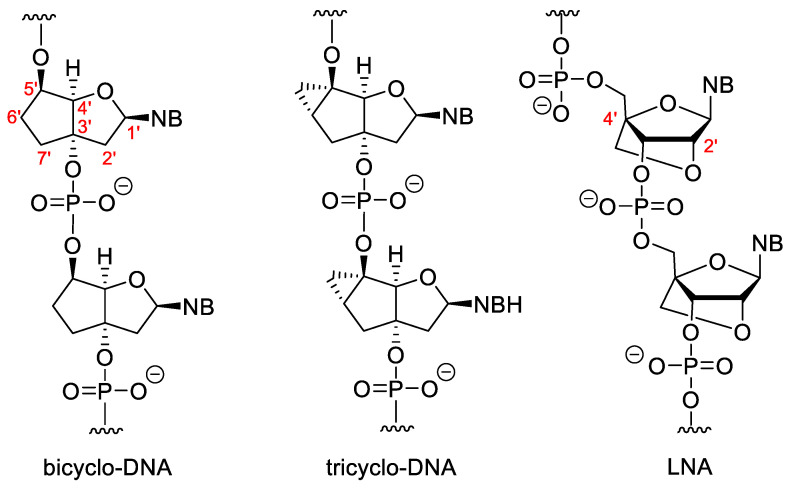
Bi- and tricyclic XNAs.

Some sugar-modified nucleoside derivatives contain bi- or tricyclic groups instead of the monocyclic ribofuranose (Figure 6). In general, these compounds show higher conformational rigidity than the mono- or acyclic NA analogues. In bicyclo-DNA, a cyclopentane ring is condensed to a tetrahydrofuran, which is connected to the nucleobase in 1′-position, while the OH groups involved in the internucleotide phosphate-ester bond are in positions 3′ and 5′. The poly-T bicyclo-DNA binds to poly-A DNA weaker than a complementary non-modified DNA oligomer. However, poly-A bicyclo-DNA hybridize to the complementary poly-T DNA stronger, than the natural poly-A DNA. The Tm of poly-A/poly-T bicyclo-DNA is higher than that of the DNA/DNA duplex with the same sequence. Bicyclo-DNA prefers the Hoogsteen and reverse Hoogsteen base pairing over the Watson–Crick [89,90].

Tricyclo-DNAs (tc-DNA) are similar to the bicyclo analogues, but in this case a third cyclopropane ring is condensed to the cyclopentane. Tricyclo DNA can form stronger duplexes with complementary DNA and RNA than the non-modified DNA/DNA or DNA/RNA duplexes, and complementary tricyclo-DNAs form very stable duplexes with each other [91]. They are very effective antisense agents. They have high serum stability and are unable to activate RNAse H [92,93]. Except for the poly-A and poly-T sequences, tricyclo-DNA prefers Watson-Crick pairing [94].

In “locked” nucleic acids (LNAs) the 2′-*O* is attached to the 4′-C via a methylene group. These dervatives are also known as 2′,4′-bridged nucleic acids (BNA).The methylene bridge fixes the furanose ring in the 3′-endo conformation that is highly advantageous for base pairing. LNA forms very stable duplexes with RNA and DNA according to the Watson–Crick rule with excellent selectivity. The incorporation of LNA monomers into DNA oligomer increases the strength of hybridization [95]. There are modified LNAs with different configurations or that contain nitrogen or sulphur in the 2′-position instead of oxygen, but the “parent” β-D-LNA possesses the most advantageous properties [96].

**Figure 7:**
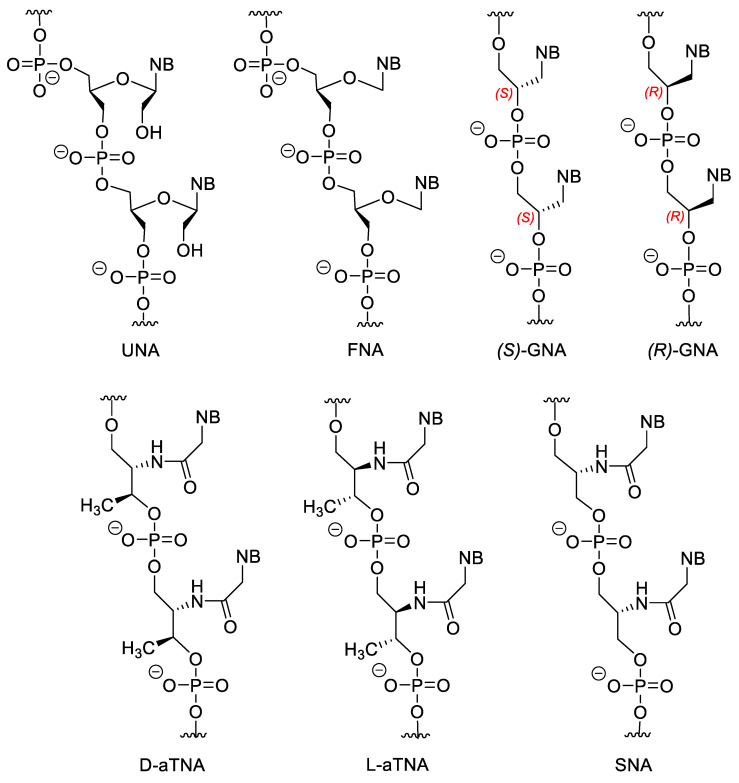
Acyclic NA analogues.

Besides mono-, and multicyclic analogues, there are acyclic XNAs, which have a more flexible backbone (Figure 7). The general advantage of these derivatives is their relatively simple and cost-effective synthesis. In the unlocked nucleic acid (UNA) the covalent bond between the 2′ and 3′ carbons is removed. They can be obtained from ribonucleosides by oxidation to 2′,3′-secodialdehydes with metaperiodate followed by reduction of the formyl groups obtained into OH-s. The incorporation of UNA monomers into DNA or RNA destabilizes the duplexes. A more interesting phenomenon is where hybridization selectivity can be influenced by incorporation of UNA monomers at the appropriate points of the oligomer. If the mismatch is located distally to the position of the UNA unit, mismatch discrimination is increased. While in case of a proximal mismatch, the discrimination is decreased especially if the mismatch can be found directly opposite to the UNA unit. This effect can be used to adjust the selectivity of the hybridization of an oligomer [97]. Even the duplex destabilizing property can be exploited, since this way the undesirable binding of the XNA oligomer to nontargeted NAs can be reduced [98].

Flexible nucleic acid (FNA) does not contain the 2′-CH_2_OH group. FNA may weakly hybridize to DNA but, interestingely, FNA monomer triphosphates can be the substrates for DNA polymerase [99,100]. Theories suggest that the first information storing molecules of life could be similar molecules to FNA [101].

Both isomers of glycol nucleic acid (GNA) can form very stable homoduplexes, and (*S*)-GNA can hybridize to RNA, however, the latter depends highly on the sequence, high G/C ratio destabilize the GNA/RNA hybride [102,103].

There are also amino-acid-based XNAs. Acyclic threoninol nucleic acid contains D- or L-threoninol instead of ribose (D-aTNA and L-aTNA). D-aTNA can form very stable homoduplexes, but cannot hybridize to DNA or RNA. While L-aTNA can bind to DNA and RNA and form homoduplexes with similar stability to the D-isomer [104,105,106]. Serinol nucleic acids (SNA) can form duplexes with DNA and RNA [106,107]. Interestingly, the chirality of the SNA oligomer, depends only on the sequence. The enantiomer of an SNA oligomer is the reverse sequenced oligomer, therefore SNAs with a symmetric sequence are meso compounds.

**Figure 8:**
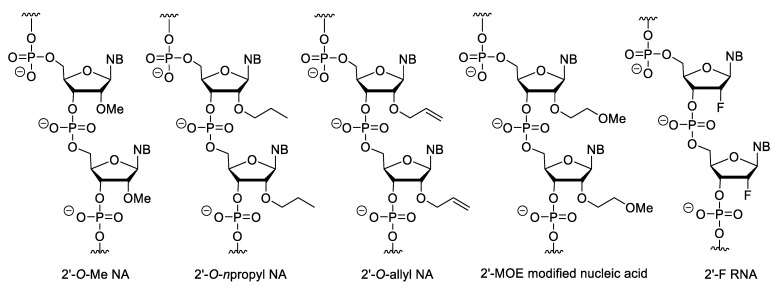
2′-Substituted nucleic acid analogues.

Minor modifications can also be made on nucleosides (Figure 8). For example, the 2′-OH of ribose can be substituted with methyl, allyl, propyl, aminopropyl and other ether groups or can be replaced by a fluorine atom (2′-F RNA) [108]. Of these modifications, the 2′-*O*-methoxyethyl (MOE) substitution is the most important because the MOE nucleic acids have the most advantageous properties and this modification is common in second generation antisense oligonucleotide (ASO) drugs. The MOE modified nucleic acids have high binding strength and nuclease resistance [109,110,111]. However, importantly, fully 2′-modified ASOs cannot activate RNase H.

### 3.3. Nucleotides with Modified Backbones

**Figure 9:**
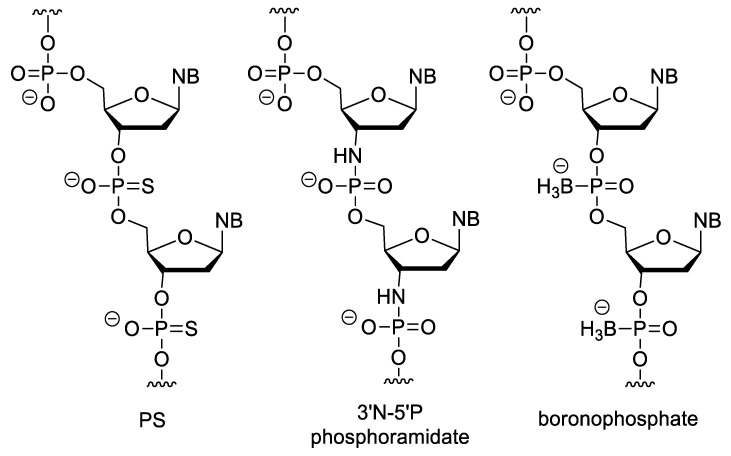
XNAs with negatively charged backbone.

In phosphorothioate (PS) oligonucleotides, one of the non-bridging oxygen of the phosphate-ester group is changed to sulphur (Figure 9). This modification retains the negative charge of the backbone. They have highly increased nuclease resistance relative to the natural nucleic acids, and RNA/PS oligonucleotide duplexes can activate RNAse H [112]. Besides the above, PSs have the advantage, that they can be easily synthesized with conventional oligonucleotide synthesis methods with a minor modification of the protocol (exchanging the oxidation step for sulfur incorporation). However, during this method, a diastereoisomeric mixture is obtained (due to the chiral nature of the PS bond, there are two isomeric forms, the Rp or endo and the Sp or exo stereoisomers) [113]. Their disadvantages include that they form slightly less stable duplexes than unmodified NAs. In addition, due to their anionic backbone, they can non-specifically bind to proteins, which can cause unwanted biological effects [114]. For example, they can activate platelets by binding to platelet-specific receptor glycoprotein VI (GPVI) [115]. PS oligonucleotides have a complex immunostimulatory effect e.g., they induce interferone synthesis and may increase the lipopolysaccharide-caused TNF-α synthesis, probably by mimicking the structure of the polyanionic glycosaminoglycans in the ECM [116]. There are also sequence-dependent effects, e.g., the CpG motif (especially the purine-purine-CG-pyrimidine-pyrimidine sequence) can stimulate B cells [117] as the CG sequence is rare and even methylated in vertebrates, while common in prokaryotes [118]. Therefore, the Toll-like receptor 9 (TLR9) of the human immune system recognizes this sequence as a bacterial pattern [119]. PS oligonucleotides strongly bind to plasma proteins and after systemic absorption, the liver and the kidney are the main sites of accumulation. In case of intravitreal administration, no significant systemic absorption was observed. Their metabolism is not primarly mediated by the CYP-450 enzyme family (which enzymes play important role in the metabolism of most xenobiotics), but by nucleases, mainly 3′-exonucleases, which cleave nucleotides from the 3′-end of oligomers. The cellular uptake of PS oligos can also be explained by their interactions with cell membrane proteins [114,120]. The PS modification is part of the first and second generation ASOs. In N3′→P5′ phosphoramidate oligodeoxynucleotides, the 3′-oxygen of nucleotides is replaced by a nitrogen. This modification increases the stability of duplexes formed with DNA and RNA. In addition, these derivatives with homopyrimidine sequences are able to form triplexes with DNA or RNA [121,122]. Incorporation of an electronegative group (fluorine) in the 2′ position of N3′→P5′ phosphoramidate oligos increases [123], while an electron donating group (OMe) in the same position decreases the stability of duplexes [124], presumably by modifying the conformation of the furanose ring. The N-methyl derivatives (3′NMe→P5′ phosphoramidate) cannot hybridize to RNA or DNA [125].

In boronophosphate oligonucleotides, one of the non-bridging oxygen of the phosphate ester is replaced by a BH_3_ group. Because of their anionic backbone, they show good water solubility, but are more lipophilic than DNA. They can be synthesized similarly to oligonucleotide synthesis, but the oxidation with iodine has been replaced by boronation of the protected H-phosphonate derivative with a borane-amine complex [122,126,127].

It is very useful to change the polyanionic phosphate ester backbone to an uncharged one because in this case the electrostatic repulsion that occurs during hybridization between the two strands is eliminated, which can cause stronger binding. In addition, due to the lack of the electric repulsion between the backbone and the negatively charged cell membrane surface, the cellular uptake can also be improved this way. The above mentioned PMOs (see Figure 5) with their phosphorodiamidate internucleotide linkages are also electrostatically neutral.

**Figure 10:**
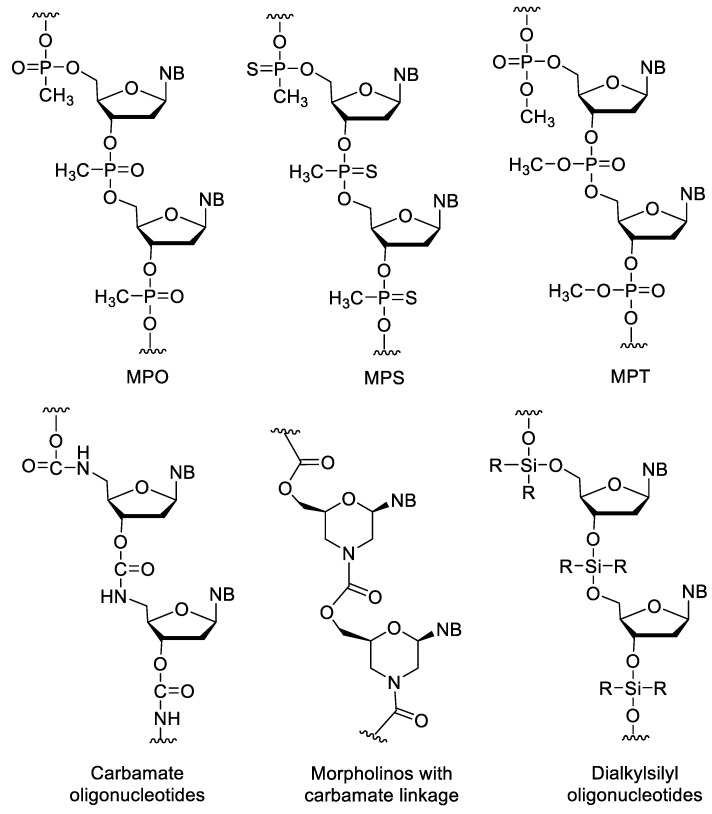
XNAs with neutral backbone.

In the alkylphosphonate oligonucleotides, one of the non-bridging oxygen of the phosphate group is replaced by an alkyl group. Among these, the methylphosphonates (MPO) are the most important (Figure 10). Their synthesis is simple by using methylphosphonoamidites in conventional oligonucleotide synthesis [128,129]. The MPOs are very resistant against nucleases. However, their affinity to natural nucleic acids is low, and they cannot activate RNAse H [122]. The incorporation of methylphosphonothioate units (MPS) into oligonucleotides slightly decreases the stability of duplexes, while significantly increasing the half-life of the oligomer [130,131,132,133,134]. Phosphotriester (PT) oligos such as methylphosphotriester (MPT) derivatives have better cellular uptake due to their neutral backbone. Inside the cell, PTs are enzymatically transformed into native oligonucleotides, so they can be used as prodrugs of siRNAs [135,136].

Carbamate (CA) oligos are stable under acidic and alkaline conditions and are resistant to some enzymes [130,137]. There are also morpholinos with carbamate linkages [138]. Dialkylsilyl oligonucleotides are acid sensitive and have very low water solubility [139,140].

**Figure 11:**
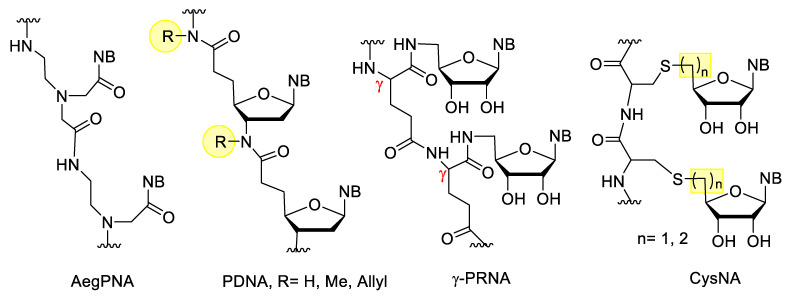
XNAs with peptide type backbones.

Peptide nucleic acids (PNAs) are very uniqe representatives of XNAs. PNA was developed in 1991 by Nielsen et al. [141]. In the original PNA, the sugar-phosphate backbone of the oligonucleotides is replaced by a poly-aminoethylglycine (Aeg), to which the nucleobases are attached through a methylenecarbonyl linker (Figure 11). This structure has several advantages, it is acyclic, achiral, electrostatically neutral, fully resistant to nucleases and highly resistant to peptidases. PNA are not sensitive to acidic or alkaline conditions. They bind excellently to DNA and RNA with high affinity and selectivity, and are also able to form triplexes with double-stranded DNA. The latter property enables PNA to anti-gene gene silencing. PNAs can be synthesized by simple peptide synthesis strategies. Their disadvantages are their limited water solubility, poor cellular uptake, and self-aggregation. The solubility decreases by increasing the length or the purine/pyrimidine ratio, but can be increased by incorporating ionic groups, and the cellular uptake can be improved by formulation or by using PNA/DNA chimeras [142]. In addition to the classic AegPNA, many other PNA derivatives have developed with a modified backbone, linker, or side chain [142,143].

Besides peptide nucleic acids, there are other XNAs with peptide bonds which also contain the ribose component. The simplest examples include the peptide deoxyribonucleic acid (PDNA) derivatives, developed by Freier et al. [144]. Integration of these derivatives into oligonucleotides increased the metabolic stability, but decreased the binding affinity. In γ-peptide ribonucleic acid (γ-PRNA) the 5′-amino-5′deoxynucleosides are linked to the γ-peptide chain through an amide bond. These derivatives are more soluble in water than PNA and due to their free 2′,3′-OH groups, their hybridization can be controlled by borax, which can form borate with vicinal diols [145,146]. Cysteinyl nucleic acids (CysNA) contain an oligocysteine backbone, to which the nucleosides are attached through a thioether bond. These can be constructed from the monomers via conventional solid phase peptide synthesis (SPPS). The monomers can be synthesized by nucleophilic substitution of the 5′-activated nucleoside and cysteine, or by thiol-ene coupling reaction between cysteine and the unsaturated nucleoside derivative [147].

**Figure 12:**
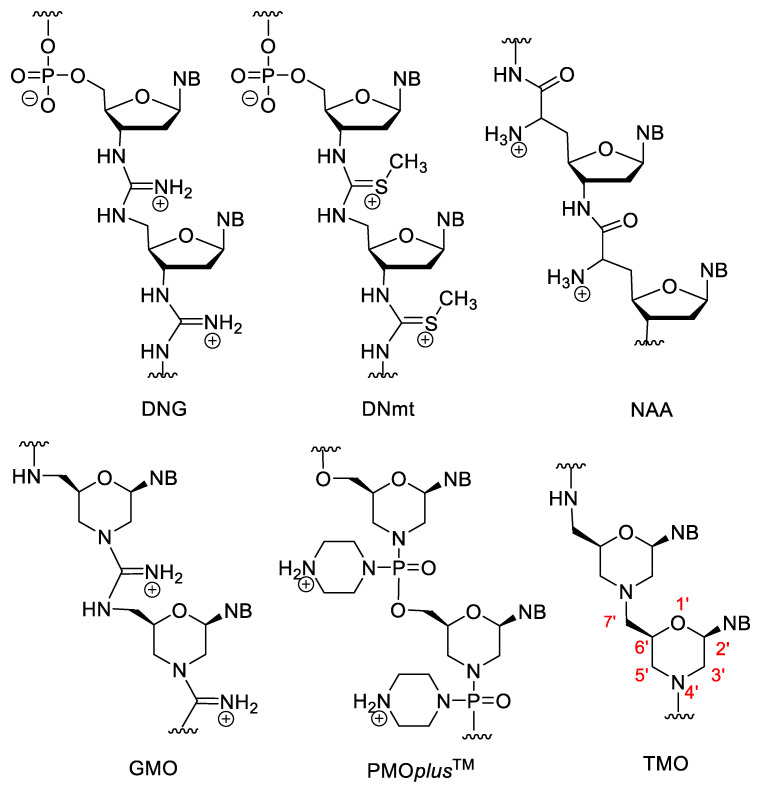
XNAs with positively charged backbone.

Besides the anionic and neutral NAs, nucleic acid analogues with positively charged backbones have also been developed. Their general advantages over anionic derivatives are their better cellular uptake, resistance to nucleases and higher binding affinity. Their theoretical common disadvantage may be their non-specific binding to proteins or to the polyanionic backbone of nucleic acids. However, in practice this is not a major problem, their sequence specificity is not decreased significantly and the XNA–protein interactions are a more serious problem for the anionic PS oligos [148,149]. The deoxyribonucleic guanidine oligomer (DNG) contains a guanidine bridge between the two nucleosides (Figure 12). DNG can form stable duplexes with complementary DNA, and poly-A or poly-T DNG can also form triplexes with the complementary DNA [148,149,150]. Relatives of DNG are the deoxyribonucleic methylthiourea (DNmt) oligonucleotides that contain an *S*-methylthiourea linker. The properties of DNmt are basically similar to the DNG. While in the case of anionic XNAs, higher ionic strength can stabilize duplexes by coating the negative charges of the backbone with cations, the stability of DNmt/DNA duplexes decreases at higher ionic concentrations [148,149,151].

Nucleosyl amino acids (NAA) are similar to the amide derivatives mentioned above, but the backbone is susbtituted by primary amino groups, which impart a cationic character to the molecule. NAA has good hybridization strength, especially the (S)-isomer, however, it has low mismatch discrimination character [149,152]. By incorporation of NAA units into DNA, zwitterionic oligonucleotides were obtained [153].

There are various positively charged morpholino oligomers. Guanidine-linked morpholinos (GMOs) have better cellular uptake than electroneutral ones [154]. In PMOplus^TM^, the nitrogen of the phosphorodiamidate group is part of a piperazine ring [84,155]. PMOplus^TM^ has been tested against various viruses, e.g., influenza virus [156]. In tightly linked morpholino oligomers (TMO), the 7′-C is directly connected to the nitrogen atom of the morpholino ring, creating a tertiary amino group, which is protonated in aqueous solution. These derivatives are synthesized by the double reductive amination-cyclocondensation reaction of nucleoside-derived secodialdehydes and 5′-amino-5′-deoxy nucleosides [157].

## 4. Oligonucleotide Drugs

After becoming familiar with artificial nucleic acid analogues, we provide a brief description of nucleic acid analogue drugs, focusing primarily on the approved ones. The active ingredients are given names that reveal the mechanism of action of the compounds. Generally, antisense oligonucleotides (ASOs) are indicated with the suffix *-rsen* (-*virsen* for antiviral drugs such as fomivirsen, afovirsen, miravirsen or trecovirsen) except for milasen because of the uniqe conditions of its naming (see below). Drugs that act on the principle of RNA interference have the suffix *-siran* (patisiran, givosiran). For aptamers, the ‘*apt*’ infix is used in the middle of the name (e.g., pegaptanib, olaptesed, emapticap, lexaptepid, etc.), however, there are rare exceptions (for example pegnivacogin) [60,158,159].

In the case of the XNAs with an anionic backbone, generally their sodium salts are formulated.

Currently, there are two approved mRNA vaccines and both have a -*meran* suffix to their name.

### 4.1. Fomivirsen

Fomivirsen (Vitravene^TM^, ISIS 2922) is a first generation, phosphorothioate type 21mer oligonucleotide analogue with a sequence of 5′-GCG TTT GCT CTT CTT CTT GCG-3′. This is the first approved gene silencing medicine (approved by the FDA in 1998 and by the EMA in 1999). It was used to treat cytomegalovirus-caused retinitis (CMV retinitis or CMVR) in patients with AIDS (acquired immune deficiency syndrome). CMV is a β-herpesvirus, which can attack mainly immunocompromised patients. The virus causes inflammation in the eye leading to vision damage or blindness [160]. The so-called intermediate-early proteins play a pivotal role in the acute infection and in the reactivation of the latent infection. These proteins are coded by the major IE gene and due to alternative splicing, several different proteins can be synthesized from this transcriptional unit. IE1 and IE2 are regulatory proteins that control the transcription of viral and host genes. IE2 is essential for virus replication [161]. Fomivirsen is complementary to part of the IE2 mRNA. Its effect consists of three components. Firstly, the sequence dependent antisense effect where there is a block of the translation from the target sequence of viral RNA, thereby reducing the level of the gene product. Secondly, there is the sequence-dependent non-antisense effect. Finally, there is the sequence-independent non-antisense effect, where, at high concentration, the PS oligonucleotides can inhibit the adsorbtion of viral particles to cells [162,163]. Fomivirsen is administered by intravitreal injection, has a half-life of ~55 h, and is degraded by nucleases. Administered this way, no systemic distribution has been detected. This decreases the potential side effects of binding to plasma proteins and interactions with systemically administered drugs. Co-administration with cidofovir is contraindicated as it may cause ocular inflammation [164]. In clinical trials, fomivirsen treatment effectively delayed the progression of CMV retinitis [165] with side effects that included local reactions (increased intraocular pressure, ocular inflammation, etc.) [166]. Due to the widespread use of effective antiretroviral therapies, CMV cases have reduced drastically, thus fomivirsen has become unnecessary, and was withdrawn in the EU in 2002 and in the USA in 2001 [167].

### 4.2. Pegaptanib

**Figure 13:**
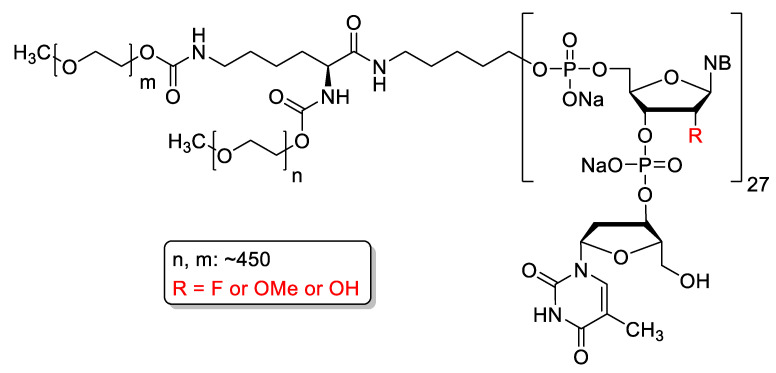
Structure of pegaptanib.

Pegaptanib (Macugen^TM^) is currently the only approved synthetic aptamer drug, used for the treatment of age-related macular degeneration [60]. Its structure is highly complex (Figure 13). A lysine is conjugated to a 28mer oligonucleotide through a 5-aminopentyl linker at the 5′ end via an amide bond, and polyethylene-glycol (PEG) monomethylethers are attached to the amino groups of lysine via a carbamate bond to improve the pharmacokinetic of the molecule. The sequence of the oligonucleotide unit is 5′-CfGmGm-AAUf-CfAmGm-UfGmAm-AmUfGm-CfUfUf-AmUfAm-CfAmUf-CfCfGm-3′-dT. The internucleotide connections are regular phosphodiester bonds, but the ribofuranose units contain various modifications. After the letter of the base, the m stands for a 2′-OMe substituent, while the f stands for a 2′-deoxy-2′-fluoro nucleoside. Thus, pyrimidine nucleosides contain a 2′-fluorine atom, while purine nucleosides contain a 2′-*O*-methyl modification except for the fourth and fifth adenosines in the sequence, which are unmodified ribonucleotides. The last thymidine at the 3′-end is linked by its 3′-oxygen to the penultimate nucleotide through a phosphate ester group. The reverse coupling of the last nucleotide to the chain protects against 3′-exonucleases [60]. Neovascular-type of age related macula degeneration (AMD or ARMD) is a degenerative disease of the eye in which neovascularization caused by vascular endothelial growth factor (VEGF) plays an important role [168]. Pegaptanib does not work on the principle of gene silencing. As its name suggests, this is an aptamer that targets VEGF. Pegaptanib is administered by intraocular injection and its side effects are mainly local reactions. Pegaptanib was approved by FDA in 2004 [33,60,169,170].

### 4.3. Mipomersen

**Figure 14:**
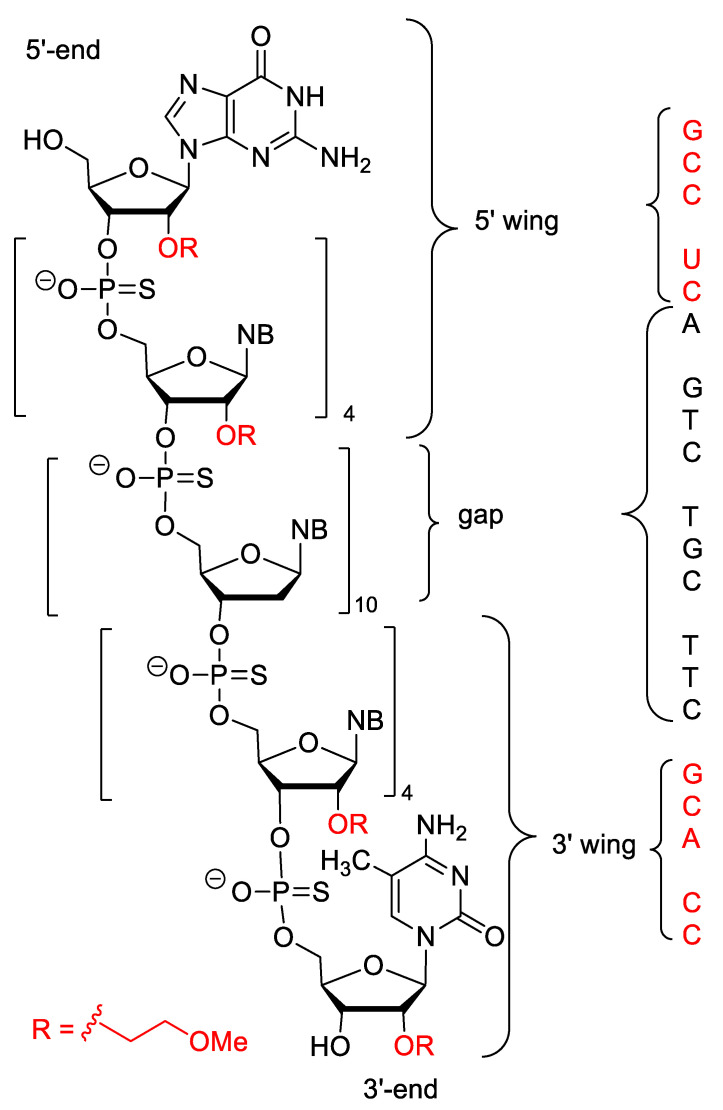
Structure of mipomersen.

Mipomersen (Kynamro^TM^, ISIS 301012) is a 20mer phosphorothioate oligonucleotide (Figure 14). It contains deoxynucleotides, except for the flanking 5-5 nucleotides on both the 5′ and the 3′ termini, which carry 2′-*O*-MOE modifications. All cytidines and the uridine in the molecule are 5-methylated, in order to increase the hybridization strength. The base sequence is 5′-GCC-UCA-GTC-TGC-TTC-GCA-CC-3′ [130]. This is a so-called gapmer structure, the flanking MOE sequences (wings) provide increased nuclease resistance for the molecule, while the central “gap” sequence is able to activate RNAse H. Mipomersen is administered by subcutaneous injection [171]. It targets the mRNA of the apolipoprotein B100 gene (ApoB-100). ApoB-100 is a protein component of LDL and other aetherogenic lipoproteines. By blocking the synthesis of ApoB-100, mipomersen can lower the level of LDL. Therefore its indication is familial hypercholesterolemia, in which the LDL level of blood is increased because of the mutation of the LDL receptor gene. Mipomersen treatment may reduce the risk of cardiovascular diseases [172,173,174]. It is the first second generation antisense drug approved by the FDA in 2013. The EMA rejected mipomersen in 2012 because of its hepatotoxicity [175].

### 4.4. Eteplirsen

**Figure 15:**
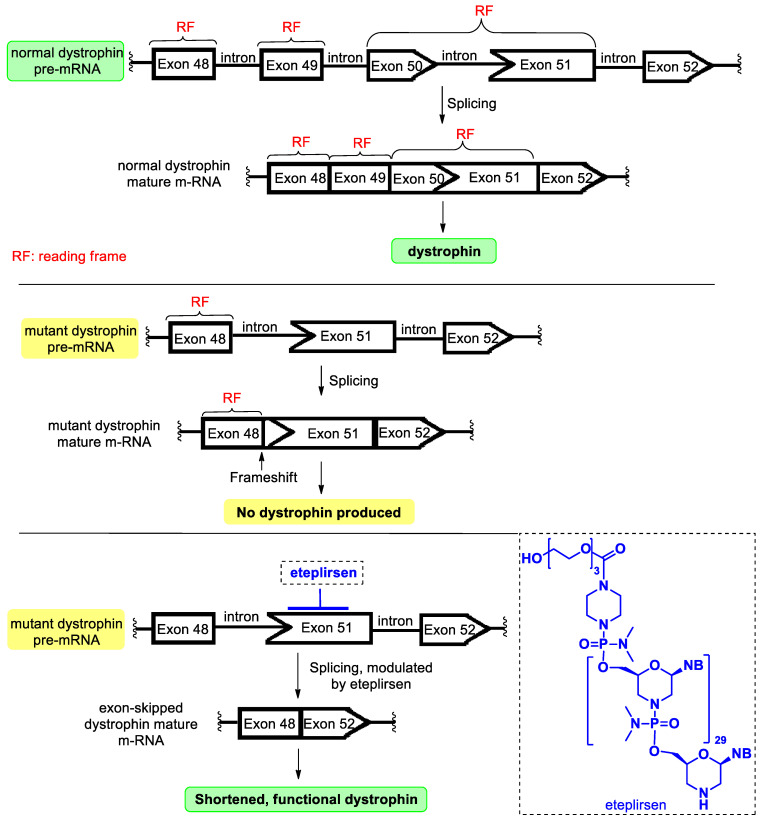
Mechanism of action of eteplirsen.

Eteplirsen (Exondys 51^TM^, AVI-4658) is a PMO derivative with the sequence 5′-CTC-CAA-CAT-CAA-GGA-AGA-TGG-CAT-TTC-TAG-3′. At the 5′ end, the phosphorodiamidate group of the oligomer is attached to a piperazine ring, while the other nitrogen atom of the piperazine is attached to a triethylene glycol motif through a carbamate linker [176]. Duchenne muscular dystrophy (DMD) is a degenerative muscle disease, caused by a mutation of the dystrophin gene on the X chromosome. There are several types of mutations that can cause this disease. One of these is the deletion of exon 49 and exon 50, a so-called frameshift mutation. This means that the reading frame of the mRNA is shifted, which causes a premature stop codon leading to early termination of protein synthesis, therefore, a small dysfunctional protein is translated from the mRNA. Without dystrophin, progressive muscle degeneration occurs, which can be fatal at a young age [176,177]. Eteplirsen works by modulating the splicing of the dystrophin mRNA. It binds to exon 51 causing its excision during RNA procession (exon-skipping). Due to the lack of exon 51, the reading frame of the mRNA is restored (Figure 15). The result of translation is a shorter, but functional protein. The protein obtained cannot work at full capacity, but an improvement in quality of life can be achieved. The condition of the treated patients is more similar to Becker type muscular distrophy (BMD, a less severe muscle disease), than to DMD [178,179]. Eteplirsen is the first PMO to be approved by the FDA (2016) [180]. However, due to limitations in the clinical trials of eteplirsen (e.g., lack of control or small sample), the EMA has rejected this drug [178,181].

### 4.5. Defibrotide

Defibrotide (Defitelio^TM^) is very uniqe among the drugs presented here. It is not a specific synthetic molecule with a well-defined structure, but a mixture of single- and double-stranded natural oligodeoxynucleotides, obtained from the controlled depolymerization of porcine intestinal mucosal DNA. The average length of the oligomers is 50 base pairs. Its resistance to nucleases is probably due to the higher order structure of the components [182]. It is used against hepatic veno-occlusive disease (VOD also known as sinusoidal obstruction syndrome—SOS), an inflammatory disease primarily associated with human stem cell transplantation (HSCT) with complex pathophysiology and various manifestations [183]. Defibrotide has complex biological effects including anti-inflammatory and antithrombotic effects. These effects are partly related to the aptameric effects of several components of defibrotide [184]. Defibrotide was approved by the FDA in 2016. The main side effects are hemorrhage and hypotension [183].

### 4.6. Nusinersen

**Figure 16:**
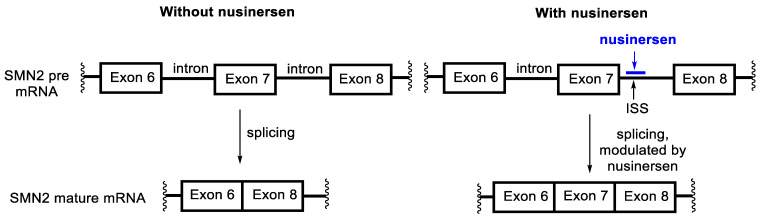
Mechanism of action of nusinersen in patient with SMA. SMA: spinal muscular atrophy, SMN2: survival motor neuron protein gene 2.

Nusinersen (Spinraza^TM^, ISIS SMN_Rx_ or ISIS 396443) is an 18-nucleotide long phosphorothioate with 2′-MOE modifications and with 5-methylated cytidines, its sequence is 5′-TCA-CTT-TCA-TAA-TGC-TGG-3′. It was developed to treat a rare disease called spinal muscular atrophy (SMA), which is the destruction of the motor neurons due to the lack of the survival motor neuron protein (SMN). SMN is coded by SMN1 and SMN2 genes. SMN2 differs from SMN1 by only one nucleotide (T instead of C), but this is enough to result in an extremly different splicing pattern in the mRNAs of the two genes. From SMN1, a mainly full length SMN (flSMN) protein is synthesized since in most cases all exons remain in the mature mRNA during the splicing. In the case of SMN2, the exon 7 is mostly missing from the mature mRNA because it is cleaved during the splicing, therefore, only a small amount of functional SMN protein is synthesized from SMN2. Nusinersen binds to one of the intron splicing regulatory regions of SMN2 pre mRNA and thereby modulates the splicing (Figure 16). As a result, the level of flSMN protein, synthesized from the SMN2 gene is increased [167,177,185]. This fascinating example illustrates the wide range of possibilities that antisense technology holds. Antisense technology was originally developed to decrease the level of the product of the targeted gene, but as the example of nusinersen shows, it can be used for the opposite purpose, too. Clinical Phase I [186] and II [187] results were published in 2016. Nusinersen was approved by the FDA in december 2016 as an orphan drug and by the EMA in 2017 [188,189]. It has only mild side effects e.g., headache [190,191]. The disadvantages of nusinersen are its high cost and the intratechal administration.

### 4.7. Patisiran

**Figure 17:**
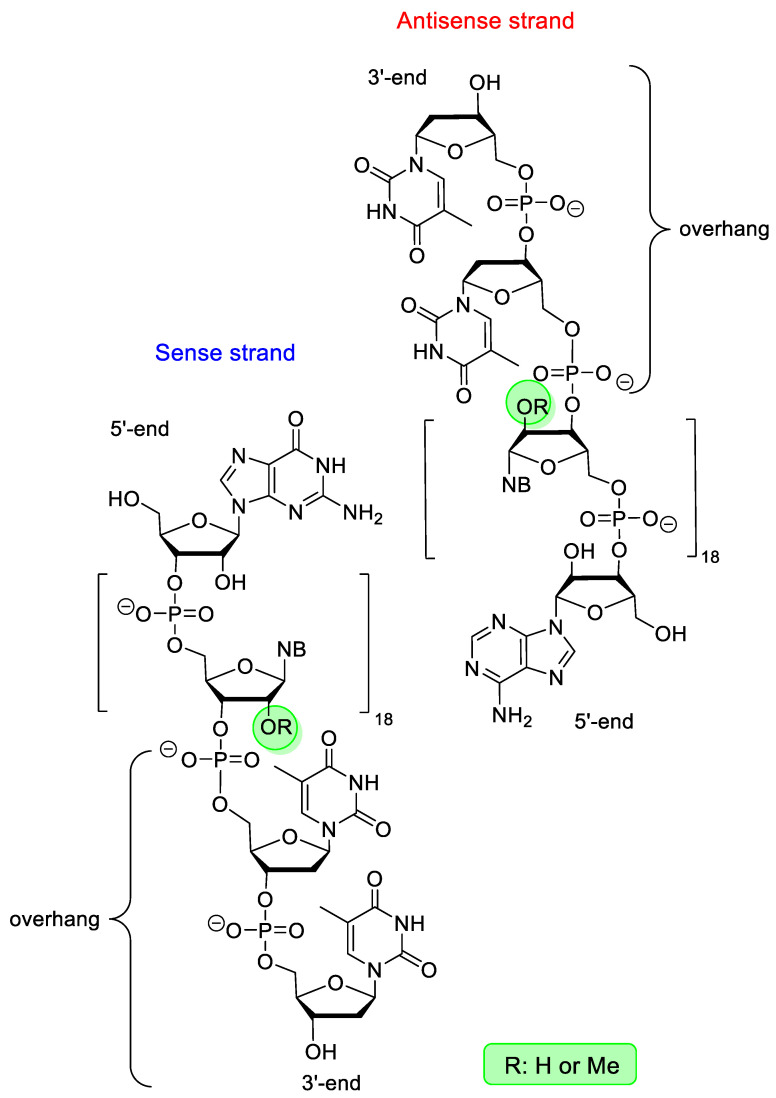
Structure of patisiran.

Patisiran (Onpattro^TM^, ALN-18328) is the first approved siRNA medicine. As described above, it is a double stranded oligonucleotide with overhanging 3′-ends (Figure 17). The sequence of the sense strand is 5′-GUmA-ACC-AAG-AGUm-AUmUm-CmCmA-UmTT-3′, and the sequence of the antisense strand is 5′-AUG-GAA-UmAC-UCU-UGG-UUmA-CTT-3′. The “m” after the letter of the base indicates a 2′-OMe substitution. All pyrimidine bases of the sense strand are 2′-OMe modified. The thymidine residues at the 3′ ends are deoxynucleotides and they form the overhanging ends, while the other monomeric units are ribonucleotides. Uniquely, patisiran contains a phosphodiester bond as the internucleotide linkage instead of PS or phosphorodiamidate [192]. Hereditary transthyretin mediated amyloidosis (hATTR) is an autosomal dominant inherited disease. Because of the mutant TTR gene, the abnormal TTR protein forms amyloid plaques primarily in the peripheral nervous system, but other organs can also be touched. The normal TTR is also involved in the deposition, therefore the mutation is dominant. TTR is mainly produced by hepatocytes, therefore patisiran is formulated with lipid nanoparticles targeting the liver. Patisiran binds to the 3′-UTR (untranslated region) of the TTR mRNA and causes its degradation via RNA interference (see more above). Main side effects are peripherial oedema and infusion-related reactions [193,194,195,196]. Patisiran was approved by the EMA and the FDA in 2018 [188].

### 4.8. Inotersen

**Figure 18:**
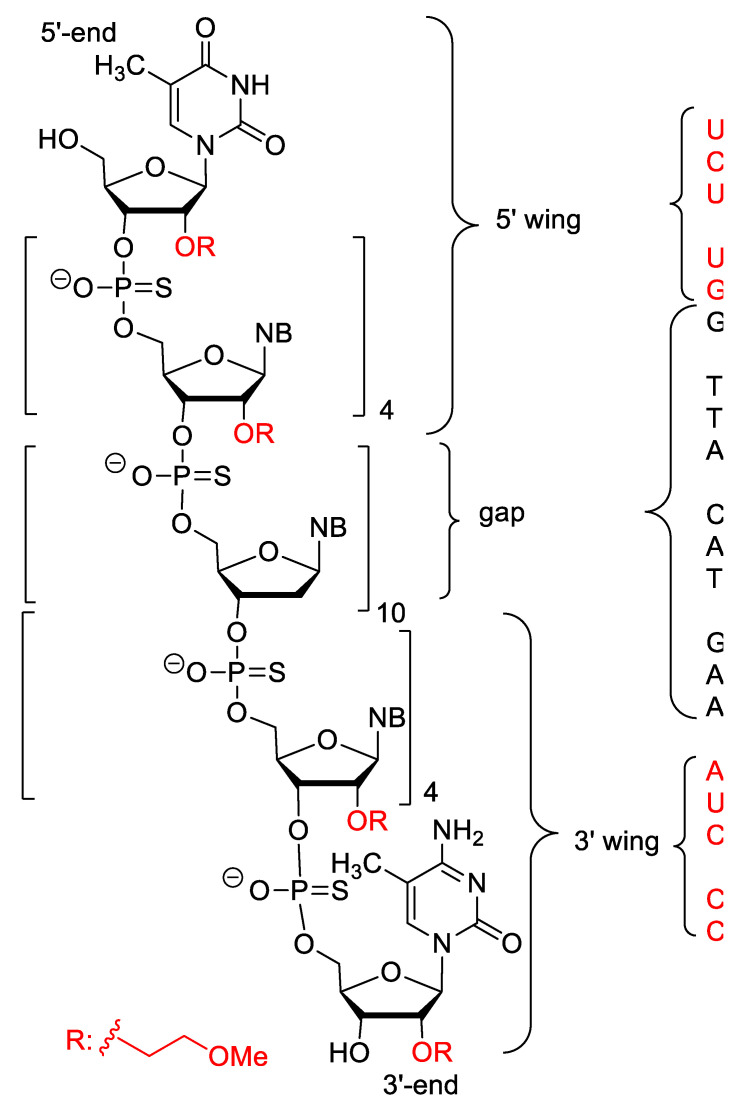
Structure of inotersen.

Inotersen (Tegsedi^TM^, ISIS-420915) is a 20-nucleotide long PS oligonucleotide with the sequence of 5′-UCU-UGG-TTA-CAT-GAA-AUC-CC-3′ (Figure 18). The pyrimidine bases are 5-methylated. It is a gapmer (similarly to mipomersen), therefore 5-5 nucleotides on the 5′ and 3′ ends are 2′-O-MOE derivatives, while the ten central monomers are deoxynucleotides [197,198]. Inotersen targets the 3′-UTR of the TTR gene, similarly to patisiran, but inotersen acts based on the antisense strategy instead of RNA interference, and therefore causes the degradation of mRNA by activating RNase H [198,199,200]. Inotersen was approved by the FDA and by the EMA in 2018 [188].

### 4.9. Milasen

Milasen is the first approved patient-customized antisense drug. It is a 22-nucleotide long phosphorotioate oligomer with 2′-MOE, modifications. As an example of its use, a child with Batten’s disease (a rare neurological disease) was hospitalized and, after sequencing her genome and examining RNA splicing, an insertion of a retrotransposon called SVA in intron 6 of the MSFD8 (also known as CLN7) gene was revealed (Retrotransposon is a DNA sequence, that is able to copy itself and integrate the copy into another location of the genome). This insertion disrupted the splicing, which led to incorrect protein synthesis. Knowing the genetic background, seven oligonucleotides were synthesized, targeting different regions of intron 6. All of these were PS oligo and six out of the seven contained a 2′-OMe modification. Finally the TY777 encoded oligonucleotide proved to be the most effective. It contained a 2′-OMOE modification instead of 2′-OMe. After animal tests, a clinical study was performed with a single patient (n-of-1 trial). Although the progression of some symptoms did not stop immediately, it slowed down. The frequency and duration of the seizures also decreased. The TY777 was named milasen after the name of the patient (Mila) [201,202,203].

### 4.10. Volanesorsen

**Figure 19:**
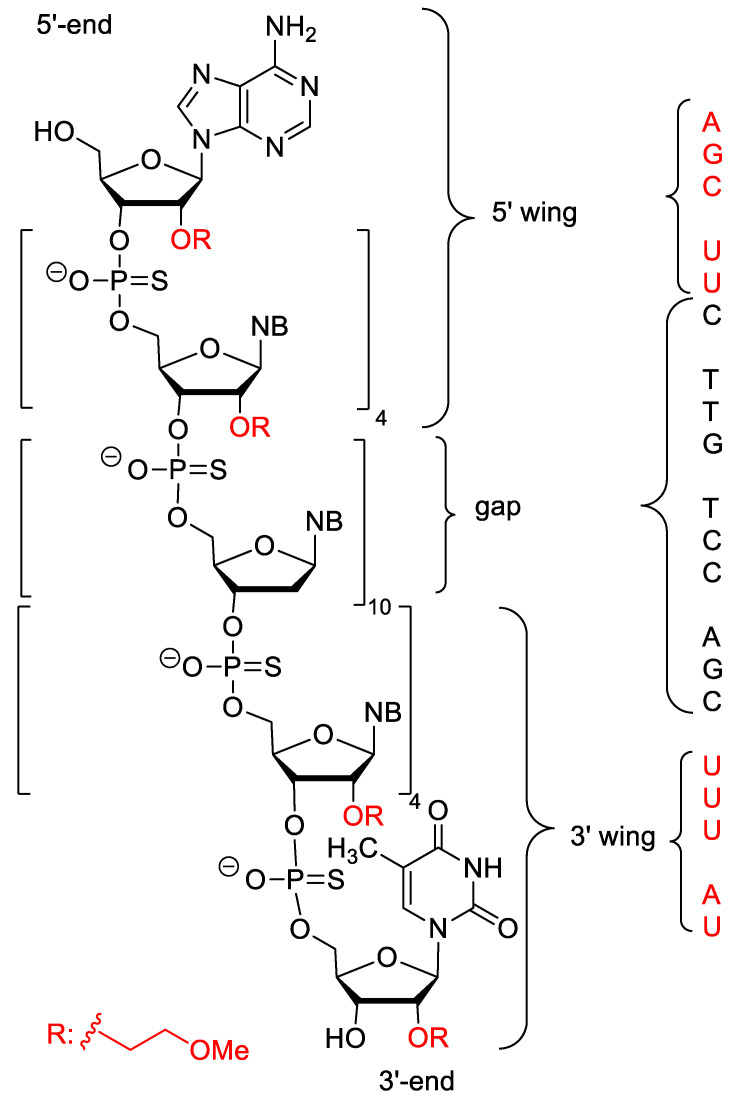
Structure of volanesorsen.

Volanesorsen (Waylivra^TM^, ISIS 304801) is a 20mer PS oligonucleotide. The sequence is 5′-AGC-UUC-TTG-TCC-AGC-UUU-AU-3′ (Figure 19). The pyrimidine bases contain a methyl group at the C-5 position. The first and last 5 nucleotides are 2′-OMOE monomers (gapmer) [204]. Familial chylomicronaemia is a disease with a high chylomicron (CM, a type of lipoproteins) level, caused by the disorder of lipid metabolism. ApoC3 is an important protein component of CM and very low density lipoprotein (VLDL) and decreases the uptake of lipoproteins. Volanesorsen targets the 3′-UTR of the APOC3 gene mRNA, causing it’s RNAse H mediated degradation and thereby reducing the plasma triglyceride level. Volanesorsen is administered as subcutaneous injection. The side effects were mainly local reactions, but a decrease in the number of thrombocytes was also observed [205,206]. Volanesorsen was approved by the EMA in 2019 [188].

### 4.11. Givosiran

**Figure 20:**
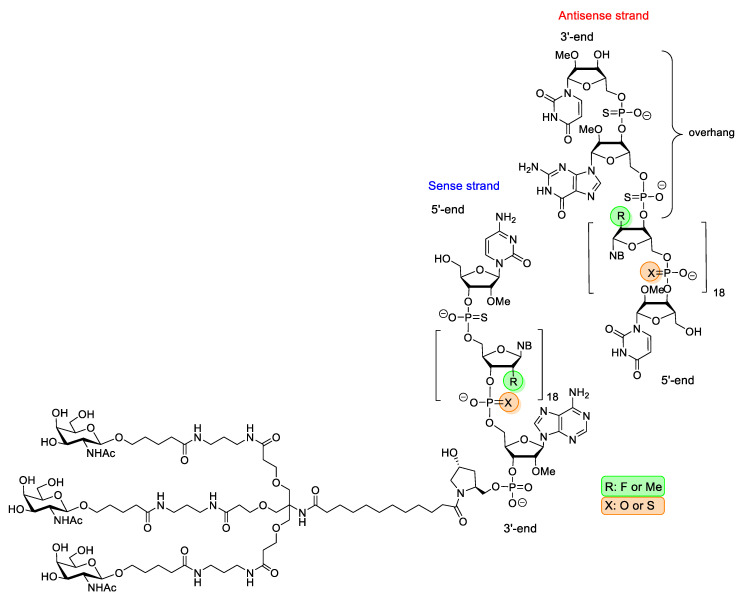
Structure of the GalNAc-siRNA conjugate givosiran.

Givosiran (Givlaari^TM^, ALN-AS1) is a double stranded oligonucleotide that acts by RNA interference. The sequence of the sense strand is 5′-CmsAmsGm-AmAmAm-GfAmGf-UmGfUm-CfUmCf-AmUmCm-UmUmAm-L96-3′. The sequence of the antisense strand is 3′-UmsGmsGm-UfCmUf-UmUfCm-UfCmAf-CmAfGm-AfGmUf-AmGfAfs-AfsUm-5′ (Figure 20). Givosiran has a very complex structure with various types of modifications. The letters “s” indicate phosphorothioate internucleotide linkages, while the other linkages are phosphodiester bonds. The “m” and “f” after the letter of the nucleobase refer to 2′-OMe and 2′-deoxy-2′-fluoro nucleotides, respectively. The antisense strand contains a 3′-overhanging region. L-96 is a trivalent *N*-acetylgalactosamine conjugate covalently linked to the 3′ end of the sense strand [207]. The function of the conjugated carbohydrate moiety is to bind to asialoglycoprotein receptors on the surface of liver cells, thereby assisting the delivery of givosiran [208]. Acute hepatic porphyria (AHP) is a disorder of the porphyrine metabolism. Levels of the δ-aminolevulinic acid (ALA) and porphobilinogen (PBG), intermediates of the porphyrine biosynthesis, are abnormally high in patients with AHP disease causing neural injury [209]. δ-Aminolevulinic acid synthase 1 (ALAS1) is responsible for the synthesis of ALA. Givosiran targets ALAS1 mRNA, causing its degradation via RNA interference. Givosiran is administered subcutaneously [208,209]. The FDA approved givosiran in 2019 and the EMA approved it in 2020 [188].

### 4.12. Golodirsen

Golodirsen (Vyondys 53^TM^, SRP-4053) is a 25mer PMO with the sequence 5′-GTT-GCC-TCC-GGT-TCT-GAA-GGT-GTT-C-3′ [210]. It contains the same 5′-modification as eteplirsen. It is used to treat DMD by causing exon skipping, similarly to eteplirsen mentioned above. However, golodirsen (as its brand name Vyondys 53 suggests) targets the exon 53 instead of exon 51. Therefore, golodirsen leads to the skipping of exon 53 and is thereby useful in the treatment of patients with a lack of exon 52 by restoring the reading frame. Golodirsen was approved by the FDA in 2019 [211,212,213].

### 4.13. Viltolarsen

**Figure 21:**
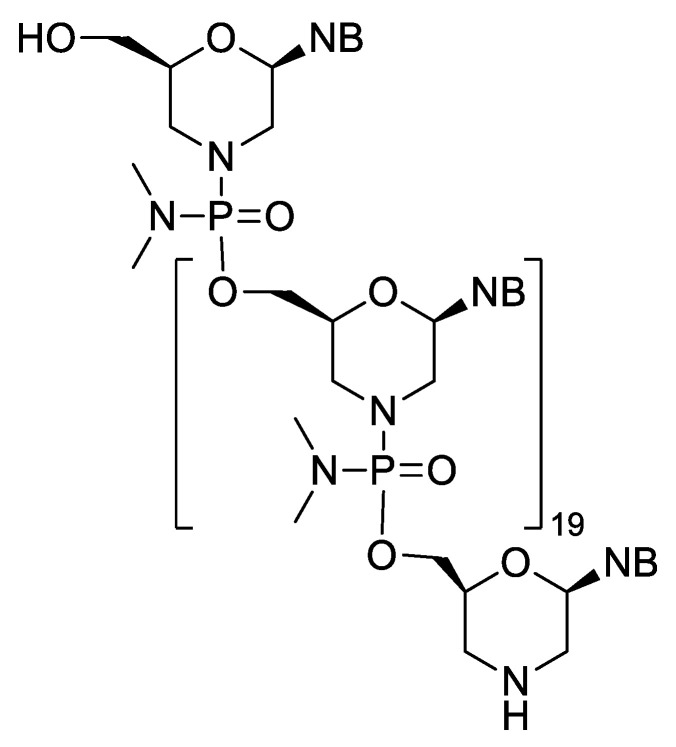
Structure of viltolarsen.

Viltolarsen (Viltepso^TM^, NS-065/NCNP-01) is a PMO with the sequence 5′-CCT-CCG-GTT-CTG-AAG-GTG-TTC-3′ (Figure 21). Unlike eteplirsen and golodirsen, viltolarsen contains an unmodified OH group at the 5′ end. It targets exon 53 of the dystrophin mRNA, similarly to the golodirsen [214,215]. Viltolarsen was approved in Japan and in the USA in 2020 for the treatment of DMD [210].

### 4.14. Inclisiran

Inclisiran (Leqvio^TM^, ALN-60212) is a double stranded oligonucleotide that acts by RNA interference. The sequence of the sense strand is 5′-Cms-Ums-Am-Gm-Am-Cm-Cf-Um-Gf-Um-dT-Um-Um-Gm-Cm-Um-Um-Um-Um-Gm-Um-L96-3′. The sequence of the antisense strand is: 3′-Ams-Ams-Gm-Am-Um-Cf-Um-Gf-Gm-Af-Cm-Af-Am-Af-Am-Cf-Gm-Af-Af-Af-Ams-Cfs-Am-5′. The letter “s” indicates phosphorothioate internucleotide linkages, while the other linkages are phosphodiester bonds. The “m” and “f” after the letter of the nucleobase refer to 2′-OMe and 2′-deoxy-2′-fluoro nucleotides, respectively. The two adenosine residue at the 3′ terminus of the antisense strand form an overhanging region [216]. Similar to givosiran, inclisiran also contains a trivalent GalNAc conjugate to help the uptake into liver cells [217]. Inclisiran targets the mRNA of the proprotein convertase subtilisin/kexin type 9 gene (PCSK9). This protein has regulatory function and decreases the number of the LDL receptors on the cell surface. Due to the lack of LDL receptors, cells are unable to take up LDL from the blood, which causes high LDL level. Inclisiran is used to treat primary hypercholesterolaemia and mixed dyslipidaemia. Inclisiran was approved in 2020 by the EMA. However, the FDA approval process was delayed because of COVID-19-related restrictions [218].

### 4.15. Lumasiran

Lumasiran (Oxlumo^TM^, ALN-GO1) is a small interfering RNA molecule. The sequence of the sense strand is 5′-Gms-Ams-Cm-Um-Um-Um-Cf-Am-Uf-Cf-Cf-Um-Gm-Gm-Am-Am-Am-Um-Am-Um-Am-L96-3′. The sequence of the antisense strand is: 3′-Ams-Cms-Cm-Um-Gm-Am-Am-Af-Gm-Uf-Am-Gm-Gm-Am-Cf-Cf-Um-Uf-Um-Am-Um-Af-Um-5′ [219]. The letter “s” indicates phosphorothioate internucleotide linkages, while the other linkages are phosphodiester bonds. The “m” and “f” after the letter of the nucleobase refer to 2′-OMe and 2′-deoxy-2′-fluoro nucleotides, respectively. Like inclisiran and givosiran, lumasiran is a GalNAc conjugate. Lumasiran is approved for the treatment of primary hyperoxaluria type 1 (PH1). During normal metabolism, glycolate is oxidized into glyoxylate by glycolate oxidize (which is coded by the hydroxyacid oxidase 1 gene = HAO1), then glyoxylate is turned into glycine, by the alanine-glycolate aminotransferase enzyme (AGT) or is converted into oxalate. In PH1, the AGT activity is reduced or lacking, which causes increased glyoxylate, and indirectly increased oxalate level. Deposition of Ca-oxalate causes serious damage in the kidney. Lumasiran targets the HAO1 mRNA and therefore decreases the glycolate oxidase activity and thereby the glyoxylate and indirectly the oxalate amount [219,220]. Lumasiran was approved in 2020 in the EU and in the USA [220].

### 4.16. Casimersen

Casimersen (Amondys 45^TM^): Casimersen is a PMO with a sequence 5′-CAA-TGC-CAT-CCT-GGA-GTT-CCT-G-3′ carrying the same modification as eteplirsen and golodirsen at 5′ position (all three compounds were developed by Sarepta Therapeutics). It targets exon 45 of the dystrophin gene, which causes the skipping of exon 45, it is therefore used to treat DMD in the cases in which the skipping of exon 45 is necessary to restore the correct reading frame. Casimersen was approved in the USA in February 2021 [221].

### 4.17. Tozinameran

**Figure 22:**
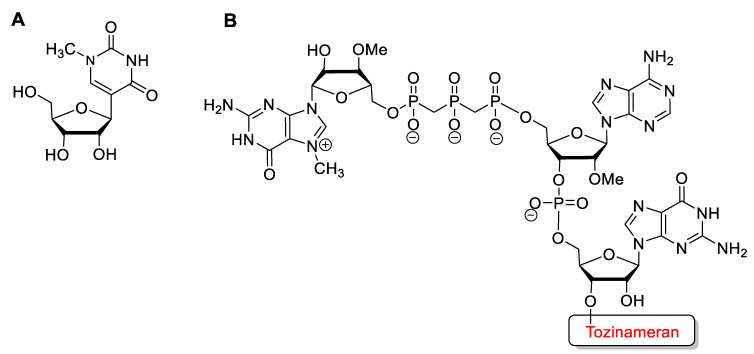
Structure of N1-methylpseudouridine (**A**) and the 5′-cap of tozinameran (**B**).

Tozinameran (Comirnaty^TM^, BNT162b2): Unlike all of the above, tozinameran is not a gene-silencing agent, nor an aptamer, but an mRNA vaccine (Figure 22). This is the newest type of oligonucleotide-based medicines. It is used to prevent coronavirus disease 2019 (COVID-19), a serious disease caused by severe acute respiratory syndrome coronavirus 2 (SARS-CoV2). COVID-19 mainly damages the lung, but other organs can also be affected. Due to the currently raging pandemic, COVID-19 is a priority problem for humanity. As the development of new medicines is a long, difficult and risky process, repurposing of the drug has come to the fore, which means the screening of existing drugs for compounds that can be used to treat COVID-19. Various drugs have been tested, e.g., chloroquine, ritonavir, ribavirin, remdesivir, molnupiravir etc. [222,223,224] but to date, breaking success is still lacking. Therefore, there is a great need for vaccine against SARS-CoV2. Spike protein is a viral structural protein, which plays an important role in viral infection by binding to ACE2 (angiotensin-converting enzyme 2), which is an early step in viral invasion of the cell [225]. Tozinameran is a 4283-nucleotide-long mRNA (counting from the 2′-*O*-Me-adenosine at the 5′-end) that encodes the spike protein of SARS-CoV2, therefore it works as an mRNA vaccine [34,226]. Tozinameran contains two mutations in its sequence (K986P and V987P, which means the change of lysine 986 and valine 987 amino acids to prolines) in order to stabilize the synthesized protein in a conformation that is more favourable for antigenicity [227,228]. Tozinameran also contains N1-methyl-pseudouridine (m1ψ) instead of uridine in order to decrease the immunogenicity of mRNA and facilitate the translation of mRNA [226]. The sequence contains a 5′-UTR (the first 53 nucleotides, derived from the human alpha-globin mRNA), the spike protein coding region (nucleotides 54-3878, with the two aforementioned mutations), a 3′-UTR which originates from 2 different mRNAs, two segmented polyA tails and a cap at the 5′-end, containing a 7-*N*,3′-*O*-dimethylguanosine and a 2′-*O*-methylsubstituted adenosine linked through a 5′-5′-triphosphate bond, to increase the stability [229]. Tozinameran received an emergency use authorisation in the EU and the USA in 2020.

### 4.18. Elasomeran

Elasomeran (Spikevax^TM^, mRNA-1273) is an mRNA vaccine, coding the spike protein of SARS-CoV2. Similarly to tozinameran, it also contains *N*-methylpseudouridine, but its exact chemical structure is currently undisclosed [228,230].

## 5. Conclusions and Perspectives

The discovery of the principle of antisense gene silencing in 1978 paved the way for the treatment of diseases at a genetic level. Over the past forty years, a number of chemically modified oligonucleotide derivatives with stunning chemical variability have been developed that have overcome the instability and other limitations of native nucleic acids, allowing the development and efficient in vivo use of genetic drugs. As a result, many nucleic acid medications for the treatment of inherited disorders and other, more common diseases have been approved, making gene therapy a reality. As is shown in Table 1, a wide range of diseases can be treated with oligonucleotide drugs from viral infections and neurological problems to metabolic disorders. Among the approved in vivo therapeutics the 2′-modified riboses, the PS oligomers and PMOs are the most common structures. There are numerous other intriguing synthetic modifications in artificial nucleic acids, which are not used in the currently approved drugs, but they can be sources of future medicines or other, not yet foreseen, applications.

Currently, antisense use of therapeutic oligonucleotides (ASOs) is the most common technique, but there is also great potential in aptamers and triantennary GalNAc-siRNA conjugates [18,37,217]. The rapid and successful development of mRNA vaccines during the COVID19 pandemic predicts the future dominance of mRNA technology in vaccine development [229,230]. In addition, the exploitation of novel approaches such as CRISPR-based genome editing may further expand the arsenal of nucleic acid therapeutics [30,231].

## Figures and Tables

**Table 1:** Nucleic-acid-based medicines approved for in vivo applications.

INN Name	Approval	Chemical Structure	Mechanism of Action	Disease
Fomivirsen (Vitravene)	FDA 1998 EMA 1999	PS	antisense	cytomegalovirus retinitis
Pegaptanib (Macugen)	FDA 2004	2′-O-Me, 2′-F, PEG-conjugate, 3′-inverted nucleotide	aptamer	age related macula degeneration
Mipomersen (Kynamro)	FDA 2013	PS, 2′-O-MOE gapmer, 5-Me-C	antisense	familial hypercholesterolaemia
Eteplirsen (Exondys 51)	FDA 2016	PMO	antisense, splicing modulation	Duchenne muscular dystrophy
Defibrotide (Defitelio)	FDA 2016	mixture of ds and ss ODNs with 50 bp average length	aptamer, complex	Sinusoidal obstruction syndrome
Nusinersen (Spinraza)	FDA 2016 EMA 2017	2′-O-MOE, PS, 5-Me-C	antisense, splicing modulation	Spinal muscular atrophy
Patisiran (Onpattro)	FDA 2018 EMA 2018	2′-OMe	RNA interference	Hereditary transthyretin mediated amyloidosis
Inotersen (Tegsedi)	FDA 2018 EMA 2018	PS, 2′-O-MOE, 5-Me-C	antisense	Hereditary transthyretin mediated amyloidosis
Milasen	FDA 2017	2′-O-MOE	antisense, splicing modulation	Batten’s disease (patient-costumized)
Volanesorsen (Waylivra)	EMA 2019	PS, 2′-O-MOE, 5-Me-C	antisense	Familial chylomicronemia
Givosiran (Givlaari)	FDA 2019 EMA 2020	PS, 2′-F, 2′-OMe, GalNAc-conjugate	RNA interference	Acute hepatic porphyria
Golodirsen (Vyondys 53)	FDA 2019	PMO	antisense, splicing modulation	Duchenne muscular dystrophy
Viltolarsen (Viltepso)	FDA 2020	PMO	antisense, splicing modulation	Duchenne muscular dystrophy
Casimersen (Amondys 45)	FDA 2021	PMO	antisense, splicing modulation	Duchenne muscular dystrophy
Inclisiran (Leqvio)	EMA 2020	PS, 2′-F, 2′-OMe, GalNAc-conjugate	RNA interference	primary hypercholesterolaemia
Lumasiran (Oxlumo)	FDA 2020 EMA 2020	PS, 2′-F, 2′-OMe, GalNAc-conjugate	RNA interference	primary hyperoxaluria
Tozinameran (Comirnaty)	FDA 2020 EMA 2020	m1ψ, 2′-OMe, 5′-cap	mRNA vaccine	COVID-19
Elasomeran (Soikevax)	FDA 2020 EMA 2020	m1ψ, 5′-cap	mRNA vaccine	COVID-19
